# Integration of Stem Cell to Chondrocyte-Derived Cartilage Matrix in Healthy and Osteoarthritic States in the Presence of Hydroxyapatite Nanoparticles

**DOI:** 10.1371/journal.pone.0149121

**Published:** 2016-02-12

**Authors:** Rupak Dua, Kristin Comella, Ryan Butler, Glenda Castellanos, Bryn Brazille, Andrew Claude, Arvind Agarwal, Jun Liao, Sharan Ramaswamy

**Affiliations:** 1 Tissue Engineered Mechanics, Imaging and Materials Laboratory (TEMIM Lab), Department of Biomedical Engineering, Florida International University, Miami, Florida, 33174, United States of America; 2 College of Veterinary Medicine, Mississippi State University, Starkville, Mississippi, 39762, United States of America; 3 Tissue Bioengineering Laboratory, Department of Agricultural & Biological Engineering, Mississippi State University, Starkville, Mississippi, 39762, United States of America; 4 Advanced Materials Engineering Research Institute (AMERI), Department of Mechanical and Materials Engineering, Florida International University, Miami, Florida, 33174, United States of America; University of Wisconsin-Madison, UNITED STATES

## Abstract

We investigated the effectiveness of integrating tissue engineered cartilage derived from human bone marrow derived stem cells (HBMSCs) to healthy as well as osteoarthritic cartilage mimics using hydroxyapatite (HA) nanoparticles immersed within a hydrogel substrate. Healthy and diseased engineered cartilage from human chondrocytes (cultured in agar gels) were integrated with human bone marrow stem cell (HBMSC)-derived cartilaginous engineered matrix with and without HA, and evaluated after 28 days of growth. HBMSCs were seeded within photopolymerizable poly (ethylene glycol) diacrylate (PEGDA) hydrogels. In addition, we also conducted a preliminary *in vivo* evaluation of cartilage repair in rabbit knee chondral defects treated with subchondral bone microfracture and cell-free PEGDA with and without HA. Under *in vitro* conditions, the interfacial shear strength between tissue engineered cartilage derived from HBMSCs and osteoarthritic chondrocytes was significantly higher (p < 0.05) when HA nanoparticles were incorporated within the HBMSC culture system. Histological evidence confirmed a distinct spatial transition zone, rich in calcium phosphate deposits. Assessment of explanted rabbit knees by histology demonstrated that cellularity within the repair tissues that had filled the defects were of significantly higher number (p < 0.05) when HA was used. HA nanoparticles play an important role in treating chondral defects when osteoarthritis is a co-morbidity. We speculate that the calcified layer formation at the interface in the osteoarthritic environment in the presence of HA is likely to have attributed to higher interfacial strength found *in vitro*. From an *in vivo* standpoint, the presence of HA promoted cellularity in the tissues that subsequently filled the chondral defects. This higher presence of cells can be considered important in the context of accelerating long-term cartilage remodeling. We conclude that HA nanoparticles play an important role in engineered to native cartilage integration and cellular processes.

## Introduction

Articular cartilage lesions in the knee frequently occur following an injury. Curl [1] demonstrated that up to 63% out of a total of 31514 knee arthroscopies suffered from chondral lesions. Another study by Hjelle [2], found that 61% out of 1000 knee arthroscopies had chondral or osteochondral lesions. Ultimately, an absence of an intrinsic healing capacity in chondrocytes prevents complete restoration of lost tissue. With limited treatment options, sustained presence of cartilage defects may lead to the onset of osteoarthritis [3–5], which will accelerate cartilage loss. Numerous studies have been done [6, 7] to treat articular cartilage defects in the knee but none of them have been able to provide consistently favorable outcomes.

Tissue engineering approaches have shown great potential in treating cartilage defects [8–10]. Cartilage tissue engineering generally involves the implantation of a degradable scaffold which supports tissue repair/regeneration processes, initiated by cells present within the defect space [11]. Since joints are highly mobile, an added requirement is the retention of the scaffold during the early phases of the regenerative process in order to promote sufficient tissue filling within the defect. We note that such retention would not only require effective integration of *de novo* cartilage with the underlying subchondral bone (in the case of osteochondral defects), but in addition, would also need to integrate well with surrounding native cartilage tissues. To permit such integration, previous studies have utilized approaches based on the principles of mechanical [12–15], chemical [16–18] and biological fixation [19–21], but with limited success.

Recently, injectable hydrogels incorporating hydroxyapatite (HA) nanoparticles have shown great potential in enhancing the integration of engineered cartilage to bone matrix [21, 22]. The targeted treatment was intended for small to medium sized osteochondral defects (< 50 mm^2^) that could arise either from an injury and/or from mild to moderate levels of osteoarthritis [23]. In addition, our recent experience suggests that HA directly promotes enhanced integration of the engineered cartilage to bone matrix by the creation of an intermediate calcium phosphate-rich transition zone, thereby permitting greater stability of the implant [22]. We note that previous studies have described that a graded transition in material properties between two different tissue matrices is an indicator of integration effectiveness [24]. On the other hand, as alluded to earlier, complete spatial integration must require fusion of the engineered cartilage to the surrounding native cartilage in addition to bone. To achieve this task, HA nanoparticles may again be useful. At first glance, the utilization of HA for cartilage-to-cartilage integration may appear counter-intuitive. However, it is important to point out that our current investigation consisted of a very small quantity (0.5% w/v ratio) of HA, with prior studies demonstrating that at these low concentrations in PEGDA, HA does not disrupt chondrogenesis within our engineered cartilage model system [22]. Thus, here we report on the utility of HA nanoparticles in promoting the integration between engineered cartilage derived from human bone marrow mesenchymal stem cells (HBMSCs) with chondral matrix secreted by healthy (HCs) and osteoarthritic (HCOAs) human chondrocytes. Based on the *in vitro* findings we subsequently transitioned our efforts towards a preliminary *in vivo* study in the rabbit model, which is also presented. A cell-free, degradable version of PEGDA was used in the *in vivo* investigation.

## Materials and Methods

Ethics Statement concerning use of Animals: Isoflurane was used to anesthetize the rabbits. Euthanization of the rabbits was conducted at the end of the in vivo portion of the study via barbituate overdose. In brief, barbituate overdose using 100 mg/ml pentabarbitol and 1 ml/10 lbs was administered by intracardiac injection. Note that, Ketamine (10 mg/kg IM) and medetomidine (0.5 mg/kg IM) was given to sedate the rabbits prior to euthanasia injection. Institutional Animal Care and Use Committee (IACUC): Mississippi State University (MSU), MSU-IACUC approval was granted on: April 9th 2014. MSU-IACUC Approval Number: # 14–029.

### Cell Culture

#### Human Bone marrow derived Mesenchymal Stem Cells (HBMSCs)

HBMSCs (Science cell, Carlsbad, CA) were seeded onto Poly D-lysine coated T-75 flasks (Fisher Scientific, Pittsburg, PA). The cells were cultured in manufacturer supplied proprietary medium (AdvanceSTEM Mesenchymal Stem Cell Expansion Kit (Thermo Scientific, Waltham, WA) until passage three (P3).

#### Healthy Human Chondrocytes (HCs)

HCs (Cell Applications Inc., San Diego, CA, USA) were sourced from non-pathologic articular cartilage. They were cryopreserved at passage 2 (P2) when shipped by the manufacturer. When the cells were received, they were plated in T-75 flasks (Fisher Scientific) and allowed to proliferate. The cells were cultured in the Human Chondrocyte Media (Cell Applications, San Diego, CA) until P4 and were subsequently used for the experiments.

#### Human Chondrocytes-Osteoarthritic (HCOA)

HCOA (Cell Applications) were isolated from the articular cartilage from patients with Osteoarthritis. They were received from the manufacturer at P1 and prior to usage in the experiments, were cultured and expanded in human chondrocyte media (Cell Applications) until P4.

### Tissue Engineered Cartilage

#### Chondrocyte-derived; Healthy

Agar is a well-established gel scaffold for culturing cartilage matrix *in vitro*. We therefore prepared engineered healthy cartilage using 2% Agar scaffold [25] in which adult chondrocytes were encapsulated. 2.5% of Agar solution (Fisher Scientific) was prepared and heated to a temperature of 70°C. Meanwhile, chondrocytes were detached from the flask using 0.25% Trypsin-EDTA (Fisher Scientific) and 20 million cells were re-suspended in 200μl of cell media. Once the Agar solution started to boil, it was removed from the hot plate and allowed to cool in a room temperature environment. When the temperature reached 40°C, 64 μl of Agar solution was mixed with 16 μl of cell suspension and casted for 5 minutes in custom designed molds (5mm in diameter and 4.1 mm in length) at the room temperature. Each gel construct consisted of a 2% Agar solution with a cellular suspension consisting of ~1.6 million chondrocytes.

#### Chondrocyte-derived; Osteoarthritic

Tissue engineered osteoarthritic cartilage was prepared in the same manner as tissue engineered healthy cartilage except for the use of HCOAs instead of HCs.

#### HBMSC-derived Tissue Engineered Cartilage

HBMSC derived engineered cartilage was prepared using our previously established protocols [22]. Procedurally, our experience has shown that the poly-D-lysine coated flasks permitted better attachment of cells initially, relative to uncoated flasks. Briefly, 15% of PEGDA solution with 0.5 w/v of HA was used to prepare the engineered cartilage. The HA nanoparticles that were used were characterized by a <200nm particle size, a surface area > 9 $\frac{m^{2}}{g}$ by the supplier (Sigma-Aldrich, Saint Louis, MO). Subsequently, 100 mg/mL of photoinitiator solution (Irgacure 2959, Ciba Specialty Chemicals, Tarytown, NY) was reconstituted in 70% ethanol and added to the monomer solution (5 μL/mL), followed by thorough stirring. This resulted in a final concentration of 0.05% w/v ratio of the photoinitiator. Next to induce polymerization, the solution was irradiated with UV light at 4–5 mW/cm^2^ for 7 minutes which we previously showed did not antagonize HBMSC viability [26].

#### Two-layer Engineered Cartilage Constructs

As stated, we first created healthy/osteoarthritic chondrocyte-derived cartilage constructs in Agar gel contained within cylindrical molds via temperature-based gelation i.e., either healthy or osteoarthritic, to mimic adjacent healthy and diseased native articular cartilage environments respectively. Next, into the same mold, HBMSCs suspended in monomer solution of PEGDA with and without HA was poured on top the Agar constructs. The PEGDA solution subsequently underwent photopolymerization to form a gel structure. Finally, a steel pin (Fisher Scientific, Catalog # 26002–10) was pierced across the two gel constructs to physically secure them for a period of 4 weeks. A physical gap would present itself at the interface of the Agar and PEGDA layers which presumably would fill with *de novo* extracellular matrix (ECM) over time resulting in a transition zone. This would permit us to assess the role of HA in contributing toward the properties of the transition zone and to the overall mechanical stability of HBMSC-derived cartilage to native/osteoarthritic chondrogenic matrix. The two layer constructs were transferred to 24-well plates, and supplied with 1 ml of chondrogenic media per sample (Fisher Scientific, Catalog # SH3088902) that were then incubated in a standard cell culture incubator operating under 95% air, 5% CO_2_, 37^°^C and humidified conditions.

Cellular constructs were similarly made without HA. In sum, four groups of 2 layers specimens were ultimately prepared as follows: 1) HCs Agar-HBMSCs PEGDA- No HA (HCs encapsulated in Agar-HBMSCs encapsulated in PEGDA without HA) 2) HCs Agar-HBMSCs PEGDA-HA (HCs encapsulated in Agar-HBMSCs encapsulated in PEGDA with HA), 3) HCOAs Agar-HBMSCs PEGDA- No HA (HCOAs encapsulated in Agar-HBMSCs encapsulated in PEGDA without HA), and 4) HCOAs Agar-HBMSCs PEGDA-HA (HCOAs encapsulated in Agar-HBMSCs encapsulated in PEGDA with HA).

## Cell Viability

To assess the viability of HCs and HCOAs encapsulated in Agar constructs, a Live-Dead assay was conducted using Calcein AM/Ethidium homodimer (Life Technologies, Carlsbad, CA) staining following the manufacturer's protocol.

In brief, we used 4.1mm long healthy/osteoarthritic chondrocytes-seeded in Agar constructs that were sectioned into 1 mm-thick slices; the slices were subsequently stained to assess cell viability. Since the depth of the constructs in later investigations represented the depth of the constructs used in the viability tests, we interpret the latter to be reflective of cell viability under the actual experimental situation. More precisely, samples from the different groups that were cultured to different time points (Day1, Day 7, Day 14 and Day 28) were as mentioned, sectioned to 1mm thin slices using a blade and immediately stained with Calcein AM and Ethidium homodimer in PBS solution and incubated for 30 minutes. Next, to remove the background fluorescence they were washed 3 times with PBS solution and then visualized under a fluorescent microscope (Olympus IX81, Olympus America Inc., Miami, FL) at excitation and emission wavelengths of 495/515 nm and 495/635 nm for Calcein AM and Ethidium homodimer respectively. Note that the viability of HBMSCs in PEGDA environment with and without the incorporation of HA was previously reported by our research group [22].

### Mechanical Testing

The interfacial shear strength between the PEGDA-based tissue engineered cartilage and the agar based healthy (or alternatively, diseased) engineered cartilage substrates were determined by performing shear testing at the interface of the 2 layers. Specifically, Interfacial shear stress mechanical testing was performed using a BOSE Electroforce 3200 mechanical testing device (Bose, Eden Prairie, MN). Data was collected real-time manufacturer software (WinTest, Bose) and was then exported to a spreadsheet (MS excel, Microsoft Corp., Redmond, WA) for additional processing. Samples to be tested (n = 6/group) were placed horizontally on the platens. One side of the sample was adhered to the upper platen while the other end was secured to the lower platen, both using super glue. The upper platen was subsequently displaced at a rate of 0.05 mm/sec with data logging conducted at every 0.03 mm of displacement. The test was ended when the 2 layers were observed to separate from each other and also accompanied with a sudden rapid decrease in the loads recorded.

### Histology

#### In Vitro Samples

Calcium and GAG matrix distribution at the interface of two layer constructs for Group 1–4 was evaluated using the Von-Kossa and Alcian Blue Stain kit (IHC world, Woodstock, MD, USA). Briefly the samples from each group were first fixed in 10% formalin. They were then embedded in molds using OCT. Sections were cut using cryostat (25 micron thickness) and were stained using the manufacturer’s protocols. In short, sectioned samples were first washed with PBS three times to remove the OCT. Then they were treated with silver nitrate solution and exposed to UV light for 60 minutes. Next, they were stained with Alcian Blue Stain, washed with distilled water and observed under a microscope to visualize both the calcium and cartilage matrix deposition at the same time on the same tissue section. A transition zone was identified as the region of *de novo* tissue fill that occurs between 2 distinct ECM layers. In the case of the histological sections, this was defined as the zone between chondrocytes in Agar representing native cartilage ECM and HBMSCs in PEGDA representing engineered tissue ECM. Initially at day zero, a physical gap or spacing would be present between the 2 ECM layers but over time (28 days), this gap would be filled with tissue manifesting itself as the ‘transition zone’.

### Energy-Dispersive X-ray Spectroscopy

To quantify the composition of elements found within the transition zone of two distinct engineered tissue matrices treated with HA, energy-dispersive x-ray spectroscopy (EDS) and EDS mapping was performed using the suggested manufacturer protocol (JEOL 6330F Field Emission Scanning Electron Microscope (FEG-SEM), JEOL Ltd., Akishima-Shi, Tokyo, Japan). The EDS was focused spatially on histological specimens containing the transition zone between engineered cartilage and engineered bone, which possessed a relatively large spatial region for assessment, thereby yielding unambiguous spectra. The purpose of the EDS analysis was to quantify the constituents of the transition zone. To obtain unambiguous spectra we assessed a relatively large spatial region sandwiched between engineered cartilage and engineered bone. To make this assessment unambiguous, an EDS map of this transition zone was also conducted.

### Explants

At 4 weeks following PEGDA with or without HA treatment, rabbit knee explants (see “2.7 In Vivo Studies” sub-section) were evaluated visually, and then placed in a tube with 10% buffered formalin Decalcification of the joint was performed using Decalcifying Solution-Lite (Sigma-Aldrich) according to the manufacturer’s protocol. The specimens were embedded in optimal cutting temperature compound (OCT; Polysciences, Inc., Warrington, PA) and sliced using a cryostat (Lecia, Buffalo Grove, IL) through the center of each defect. Samples were subsequently stained with hematoxylin and eosin (H&E) according to manufacturer’s protocol (ScyTeck Laboratories, UT) and viewed under a microscope (Amscope, Irvine, CA) to visualize cell and repair tissue morphology within the cartilage defects. For quantification purposes, cell counts were performed on the images of the H&E sections (n = 6 defects for microfracture + HA; n = 3 defects for microfracture alone) to quantify the number of cells present in the repair tissue (ImageJ, 1.48v, National Institutes of Health, Bethesda, MD).

### Quantitative Real Time-Polymerase Chain Reaction q(RT-PCR)

To assess the gene expression of HBMSC-derived, *in vitro grown* cartilage, quantitative real time-polymerase chain reaction (qRT-PCR) analysis was conducted at the interface location of stem cell and chondrocyte-derived engineered cartilage as well as at a spatial distance 4 mm from the interface, well within the HBMSC-derived *de novo* tissue region. First, engineered cartilage derived from HBMSCs after 28 days of culture was extracted from each group.

Each sample was subsequently cut into 2 parts. One section comprised of the transition region of 1 mm, which included the interface location while the remainder consisted solely of engineered tissue derived from HBMSCs. Three samples from each group were cut in a similar fashion and, crushed and pooled together for analysis. This was repeated for another 2 samples from each group, i.e., n = 3 samples for each of the interfacial and distal cartilage locations. Total mRNA was extracted from each group three times (SV total RNA isolation kit, Promega, Madison, WI). RNA isolation, reverse transcription and the qRT-PCR was subsequently performed as previously reported [22, 27, 28]

The primers (Table 1) were designed using the Basic Local Alignment Search Tool (BLAST) program, National Center for Biotechnology Information (NCBI) to amplify the target sequences. These markers were chosen to assess the quality of *de novo* cartilage [29–33]. In order to interpret the results, the change in cycle threshold (Cт) values were averaged and normalized with GAPDH using the ΔΔCт method [34]. Fold changes were calculated as 2^- ΔCт^, and the gene expression ratio was plotted.

**Table 1:** Quantitative RT-Polymerase Chain Reaction Primer Sequences.

Genes	Forward Primer	Reverse Primer
GAPDH	AATGAAGGGGTCATTGATGG	AAGGTGAAGGTCGGAGTCAA
Aggrecan	GCGAGTTGTCATGGTCTGAA	TTCTTGGAGAAGGGAGTCCA
SOX9	GTAATCCGGGTGGTCCTTCT	GTACCCGCACTTGCACAAC
Collagen II	AGACTTGCGTCTACCCCAATC	GCAGGCGTAGGAAGGTCATC
MMP 13	ACATCCCAAAACGCCAGACAA	GATGCAGCCGCCAGAAGAAT
Runx2	AATCCTCCCCAAGTTGCCA	TTCTGTCTGTCCTTCTGGGT
Type X Collagen	TGGATCCAAAGGCGATGTG	GCCCAGTAGGTCCATTAAGGC
Type I Collagen	TGAGAGACCAAGAACTG	CCATCCAAACCACTGAAACC
Osteocalcin	CACTCCTCGCCCTATTGGC	CCCTCCTGCTTGGACACACAAAG

### In-Vivo Studies

To further evaluate the utility of HA in promoting engineered to native cartilage tissue integration an *in vivo* study was conducted following institutional animal care and use committee-IACUC approval (approval #: 14–029). Three mature (4kg, 6 months) New Zealand white rabbits (Oryctolagus cuniculus) were utilized. The animals were endotracheally intubated and a surgical plane of anesthesia was maintained with inhaled isoflurane. Both hind legs were clipped and prepared for aseptic surgery. Access to the stifle joint was gained by a medial parapatellar incision and lateral dislocation of the patella bilaterally. During the first surgery, bilateral cranial cruciate ligament transection was performed with a #11 scalpel blade and the incisions was closed in layers (fascia, subcutaneous tissue, and skin) using 3–0 PDS and 4–0 Monocryl sutures in order to induce osteoarthritis.

Two weeks following this surgery, the animals underwent a second surgical procedure in which 4 mm chondral defects (3 per stifle) were created within the femoral trochleas. The defects were treated with either PEDGA alone or with PEDGA + HA. Of note, the PEGDA utilized for these *in vivo* studies was a degradable counterpart (HyStem-C Hydrogel Kit, ESIBIO Stem Cell Solutions, Alameda, CA) to the one used in the *in vitro* investigations. However the amount of PEGDA and HA utilized was identical to the concentrations used *in vitro* (15% w/v PEGDA and 0.05% w/v HA).The subchondral bone beneath the chondral defects was micro-fractured using an eighteen gauge hypodermic needle to induce bleeding. Finally, the incision was closed in layers (fascia, subcutaneous tissue, and skin) using 3–0 PDS and 4–0 Monocryl sutures. The rabbits were continuously observed until they recovered from anesthesia and were euthanized four weeks following the second surgery.

### Statistics

Statistical analysis was performed for the *in vitro* results obtained from the cell viability, interfacial shear strength testing and qRT-PCR outcomes. The results were reported as mean ± standard deviation. Commercially available software (SPSS, IBM, version 20, Armonk, NY, USA) was used to perform the statistics. To compare means and to determine statistically significant differences between groups, a one way ANOVA and post hoc Tukey test was used. In cases where only two groups were compared, t-test for independent groups was utilized. This was also the case for the in vivo-derived specimens, wherein an independent t-test was conducted (SPSS, IBM) to assess if a significant difference (p < 0.05) was present in terms of cell counts, between the HA treated versus untreated group (n = 3); results were reported as the mean ± standard error of the mean. In all cases, statistical differences between groups were determined to be significant if p < 0.05.

## Results

### Cell Viability

**Fig 1:**
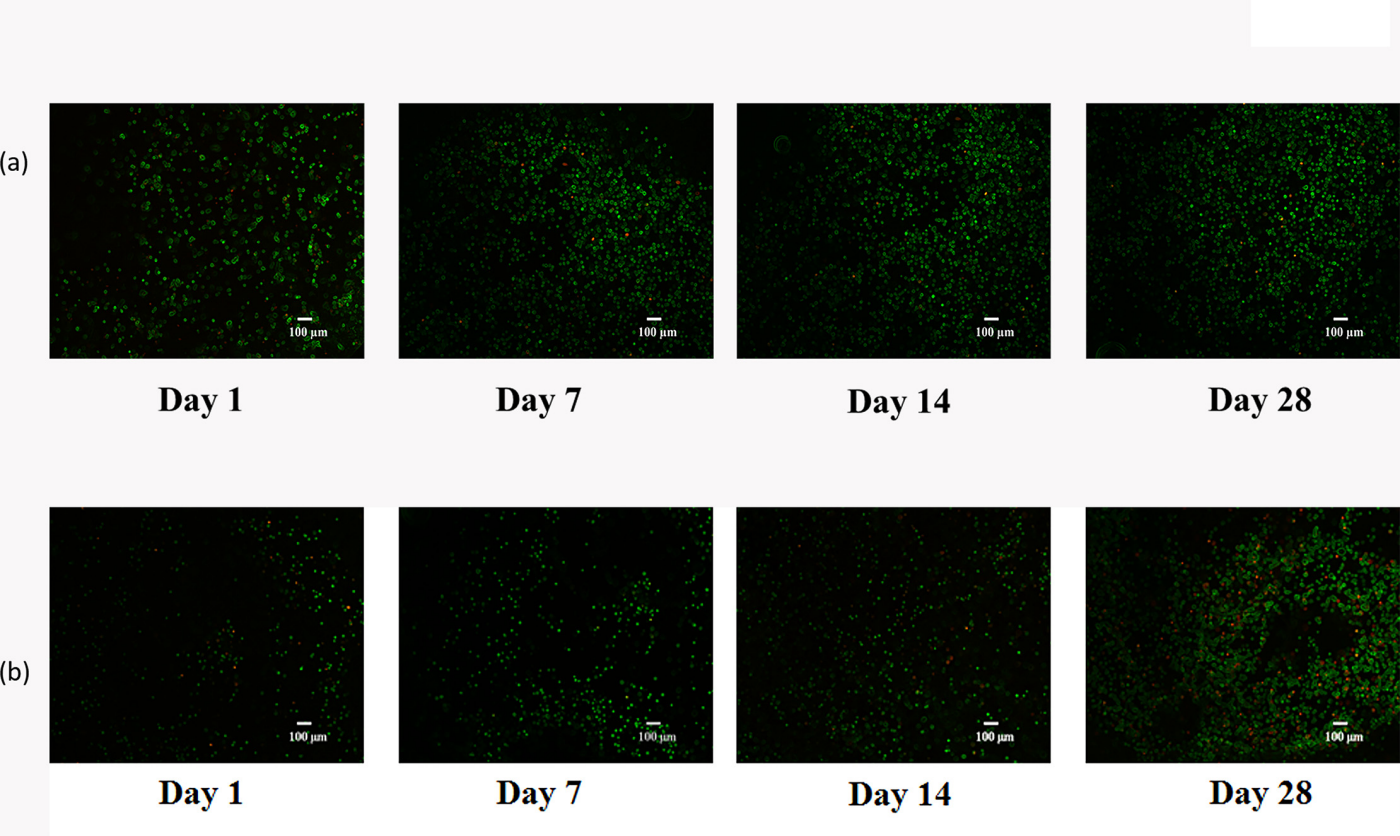
Viability assessment. (a) healthy and (b) osteoarthritic chondrocytes during 28 days of culture. We found that ~ 97% of the HCs and ~ 86% of the HCOAs were viable in the Agar gel at 28 days of growth.

The viability of the cells in the engineered scaffolds was observed over a period of 28 days at 4 time points (Day 1, Day 7, Day 14 and Day 28). We found that ~ 97% of the HCs and ~ 86% of the HCOAs were viable in the Agar gel at 28 days of growth (Fig 1).

### Mechanical Testing

**Fig 2:**
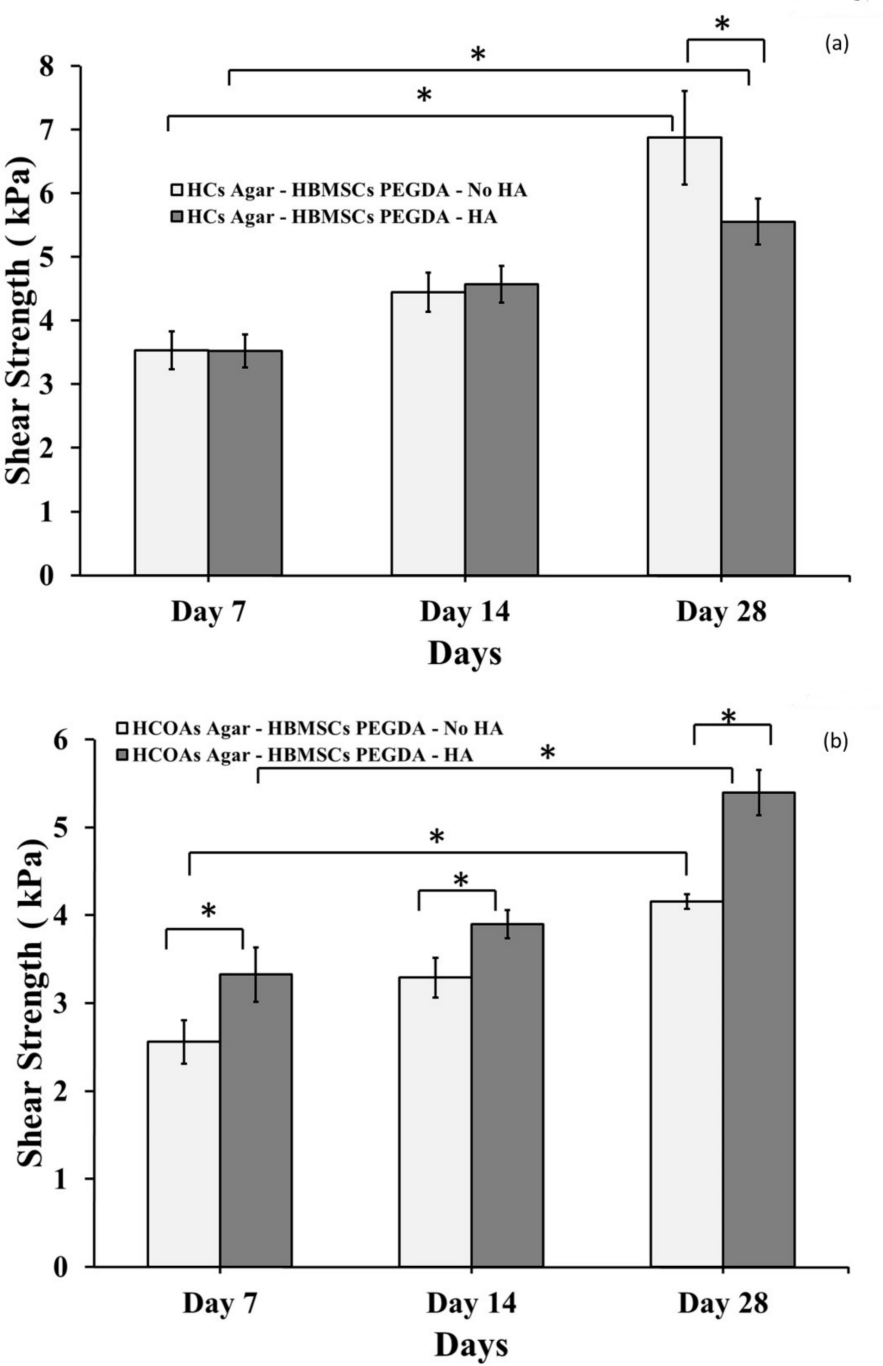
Interfacial shear stress. (a) Tissue engineered cartilage integrated with healthy cartilage mimics with and without the presence of HA. (b) Tissue engineered cartilage integrated with osteoarthritic cartilage mimics with and without presence of HA. The “*” indicates that the difference between the groups was statistically significant (p < 0.05).

We found a significantly higher shear strength (p<0.05) in HCs Agar-HBMSCs PEGDA- No HA compared to HCs Agar-HBMSCs PEGDA-HA after both 7 and 28 days of tissue culture (Fig 2(A) and S2 File). On the other hand, at all-time points (Day 7, Day 14 and Day 28) the HCOA-based samples with HA nanoparticles exhibited significantly higher shear strength (p<0.05) when compared to the corresponding group without HA (Fig 2(B) and S3 File).

### Histology

#### In Vitro Samples

**Fig 3:**
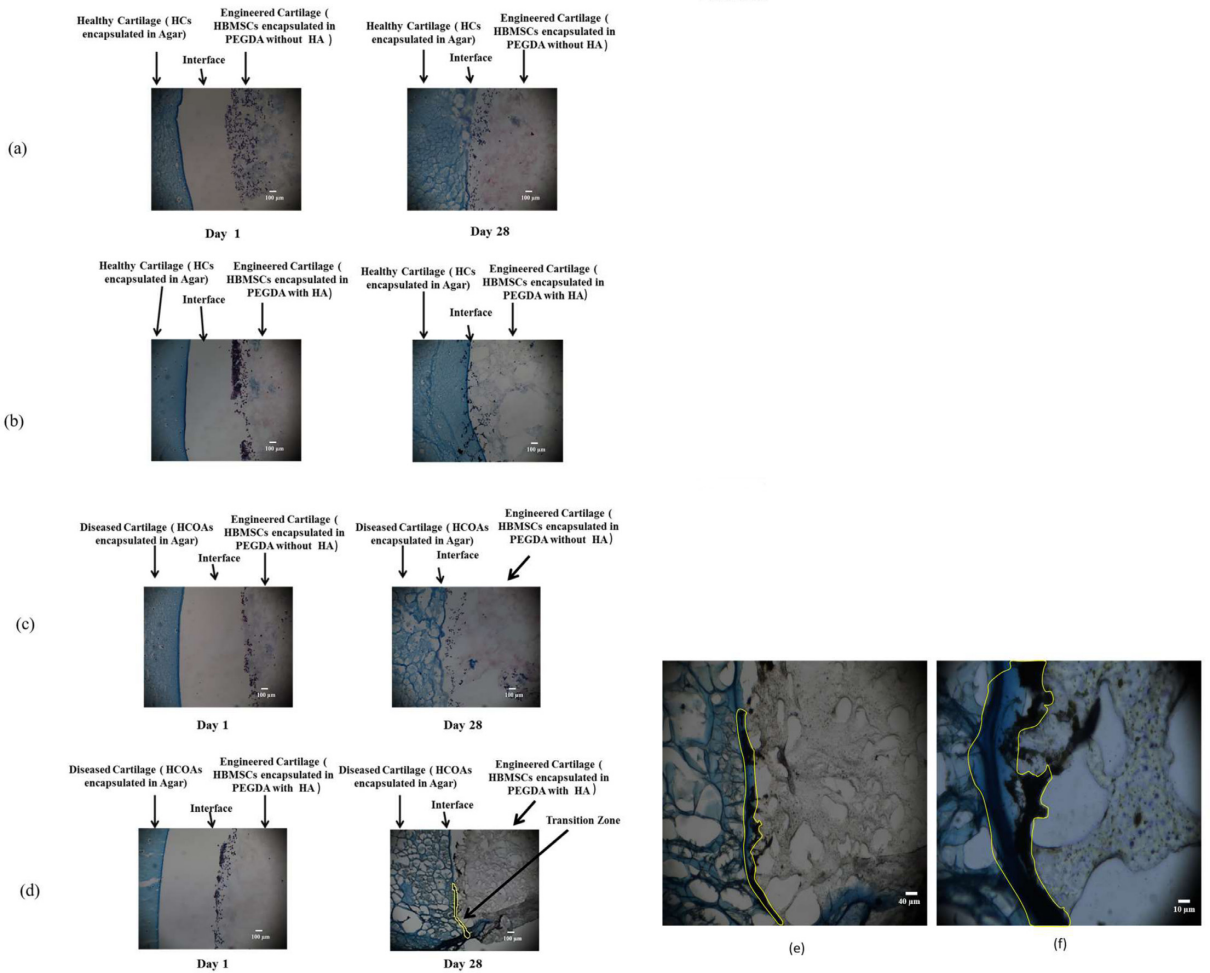
Von Kossa—Alcian Blue histology. (a) Tissue engineered cartilage derived from HBMSCs integrated with HC-secreted cartilage matrix without HA at Day 1 and at Day 28. (b) Tissue engineered cartilage derived from HBMSCs integrated with HC-secreted cartilage matrix with HA at Day 1 and at Day 28. (c) Tissue engineered cartilage derived from HBMSCs integrated with HCOA-secreted cartilage matrix without HA incorporation at Day 1 and at Day 28. (d) Tissue engineered cartilage derived from HBMSCs integrated with HCOA-secreted cartilage matrix with HA incorporation at Day 1 and at Day 28—Progressive filling of the transition zone with calcium phosphate deposits (indicated by dotted yellow lines) in the group with HA was found to occur. (e) A zoom-in of the transition zone (additional 2.5X that of Fig 3(d)). (f) Even further zoom-in of the transition zone (additional 10X that of Fig 3(d)).

Histological sections revealed that HCs encapsulated in Agar-HBMSCs encapsulated in PEGDA with HA did not form a transition zone, but instead presented with a physical spacing or gap between the two engineered constructs (Fig 3(B)). By transition zone, we refer to a defined spatial region located between the tissue engineered two-layer constructs (e.g. between HBMSCs engineered cartilage and HCs / HCOAs engineered cartilage), that is made up of heterogeneous components derived from the two layers and is relatively much larger than the size of the actual interface. However regardless of whether or not HA was present, a narrowing of the gap was observed to have occurred in the constructs at 28 days in comparison to samples evaluated after 1 day of tissue culture (Fig (3A and 3B).)

After 28 days of tissue culture, in HCOAs encapsulated in Agar-HBMSCs encapsulated in PEGDA with HA, a thin transition zone was formed between HBMSC-derived engineered cartilage and HCOA-derived cartilage; on the other hand, when HA was not utilized, only a narrowing of the gap between the two layers was observed as was previously seen in the HC groups (Fig (3C, 3D, 3E and 3F)).

#### EDS

**Fig 4:**
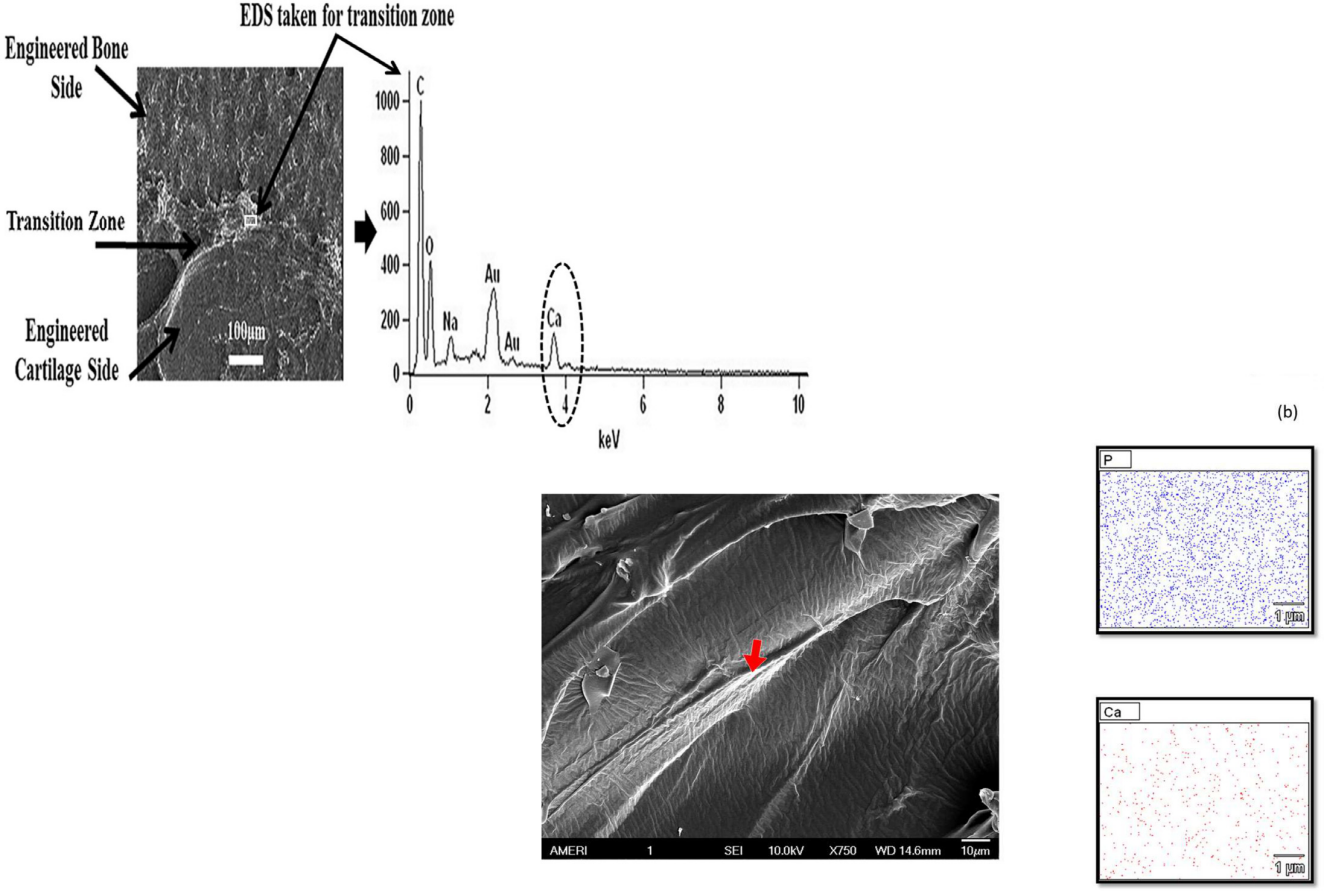
Energy-Dispersive X-ray Spectroscopy EDS and its map. (a) Elementary composition of the transition zone using (EDS). A large transition region between engineered cartilage and engineered bone treated with HA was analyzed after 28 days of culture. Elemental Calcium in the order of ∼ 6.41% was found to be present in the transition zone. (b) EDS map of the transition zone with the revelation of both robust presence of phosphorous and calcium, the two primary constituents of HA. The map thereby confirmed HA’s presence within the transition zone.

EDS analysis in the transition zone between *de novo* cartilage and bone matrix for elemental Calcium was found to be approximately 6.41% (Fig 4(A)). The EDS map (Fig 4(B)) provided clarity on the spatial location of the transition zone at 28 days following tissue culture, with distinct brighter contrast relative to the surrounding ECM, with additional magnifications. Moreover, the map confirmed the presence of both Calcium and Phosphorus elements, both constituents of HA.

#### Explants

**Fig 5:**
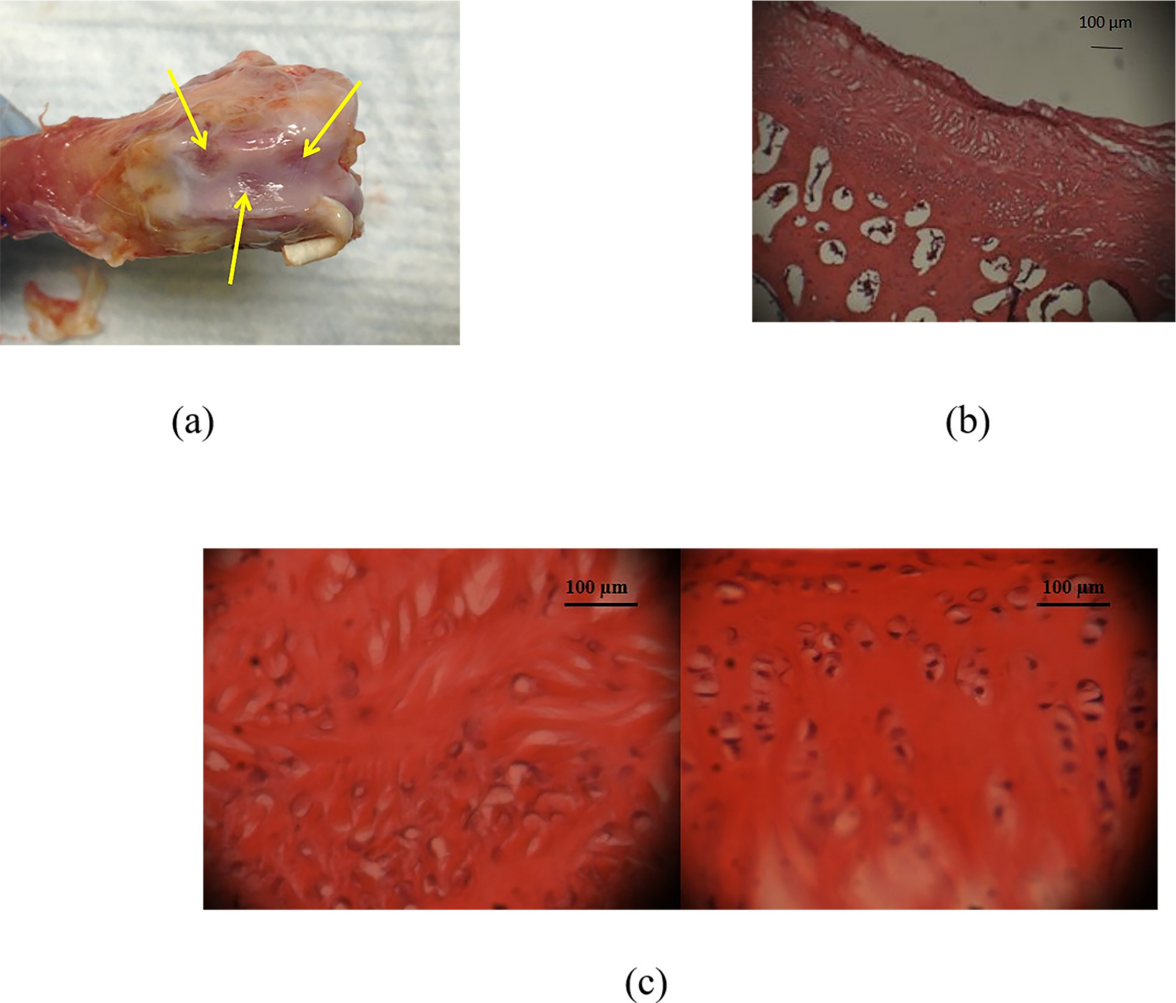
Bone Morphology. (a) Macroscopic view of bone joint morphology. Three chondral defects per rabbit knee were created in the trochlear groove, shown by the yellow arrow with microfracture of the subchondral bone. (b) H&E staining (40X magnification) of chondral defect (c) H&E staining (250X magnification) of: (Left) without the incorporation of HA and (Right) with HA incorporation. The inclusion of HA nanoparticles promoted improved cellular organization within the newly formed tissue in the defect.

Gross morphology as well as histology suggested robust tissue filling of defects treated with PEGDA-alone as well as those treated with PEGDA+HA (Fig 5). At 4 weeks following PEGDA with or without HA treatment, the average cell count in rabbit chondral defects with PEGDA treatment-alone was found to be 28.7 ± 16.2 cells. On the other hand, defects treated with PEGDA+HA had significantly higher (p < 0.05) cell counts, in the order of 72.3 ± 8.5 cells.

### Quantitative Real-Time Polymerase Chain Reaction (qRT-PCR)

**Fig 6:**
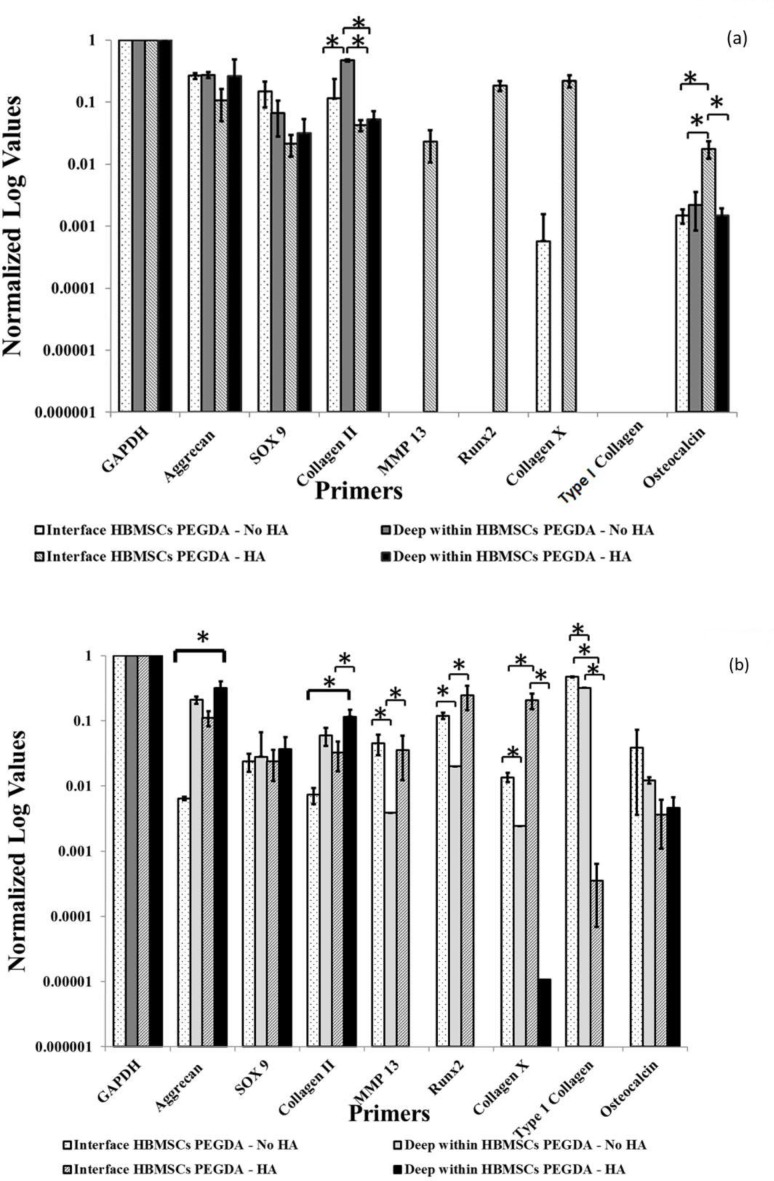
Quantitative Real Time-Polymerase Chain Reaction. (a) q(RT-PCR) of HBMSCs derived engineered cartilage integrated to HC-secreted cartilage matrix at a region of 1 cm (proximal) and 4cm (distal) from the interface within the HBMSC construct. In samples both with and without HA, a high expression of Aggrecan, SOX9 and Collagen Type II at both the proximal and distal positions was found, indicative of a healthy articular cartilage phenotype. In addition, in the samples with HA, high gene expression of MMP13, Runx2 Collagen Type X and significant expression of Osteocalcin (p < 0.05) at the proximal location was found. The absence of these bone ECM genes thus preserved the cartilage phenotype when HA was not incorporated in healthy engineered cartilage matrix. (b) q(RT-PCR) of HBMSCs derived engineered cartilage integrated with HCOA-secreted cartilage matrix at a region of 1 cm (proximal) and 4cm (distal) from the interface within the HBMSC portion. In samples with HA, a significantly lower expression of Collagen Type I (p < 0.05) at the proximal location was found, i.e., preservation of the articular cartilage phenotype (while still maintaining robust expression of Aggrecan, SOX9 and Collagen Type II). In the specimens with HA, distal locations deep within the HBMSC-derived engineered tissue did not or negligibly expressed MMP13, Runx2 and Collagen X (p < 0.05). We speculate that the presence of HA at the interface and the subsequent creation of a calcium phosphate-rich transition zone served to potentially further reduce the spread of osteoarthritic conditions to the *de novo* cartilage formed by the HBMSCs. Note that the expression of MMP13, Runx2 was completely absent in specimens with HA in the deep zone (hence bar not shown), but these findings were nonetheless significantly lower in expression (p < 0.05) compared to the expression of these genes in specimens without HA.

After 28 days of culture in chondrogenic media, it was observed that HBMSCs derived tissue engineered cartilage in HCs Agar-HBMSCs PEGDA- No HA, exhibited relatively high expression of Aggrecan, SOX9 and Collagen Type II genes in both the proximal and distal regions (relative to the interface with HCs-derived engineered cartilage; Fig 6(A) and S5 File). On the other hand, there was a higher gene expression of MMP13, Runx2, Collagen X and in particular, significant expression of Osteocalcin (p < 0.05) at spatial locations proximal to the interface in HBMSCs derived tissue engineered cartilage within HCs encapsulated in Agar-HBMSCs encapsulated in PEGDA with HA (Fig 6(A) and S5 File).

In the HBMSCs derived cartilage from HCOAs Agar-HBMSCs PEGDA-HA group, it was found that there was significantly lower gene expression (p < 0.05) of Collagen Type I in both the proximal and distal regions from the interface (Fig 6(B) and S6 File). Yet, a robust expression of articular cartilage markers, Aggrecan, SOX9 and Collagan Type II was maintained. In contrast, gene expression of Type I collagen was significantly higher (p < 0.05) at both the interface and deep locations in the HBMSCs derived engineered cartilage within HCOAs encapsulated in Agar-HBMSCs encapsulated in PEGDA without HA samples. Moreover, the specimens with HA did not or negligibly expressed bone markers MMP13, Runx2 and Collagen X compared to without HA counterparts in osteoarthritic environments; the comparisons between these groups were found to be significantly different (p < 0.05; Fig 6(B) and S6 File).

## Discussion

Over the past decade, studies have focused on augmenting the integration of engineered cartilage to bone [35, 36] to establish a more stable implant. However of equal importance is the need for effective integration of the engineered cartilage with surrounding native articular cartilage, especially in the case of focal chondral defects. We thus sought to build on our previous work [22] on enhanced integration between cartilage and bone matrix using HA in a similar manner except here, we examined the utility of HA nanoparticles in promoting the anchorage and integration of engineered cartilage to healthy as well as osteoarthritic cartilage ECM. Note that our previous work [22] already demonstrated that the relatively small HA concentration (0/5% w/v) used in the engineered cartilage model system did not disrupt the development of *de novo* cartilage. In the current study, we started with an *in-vitro* investigation, in which a well-characterized non-degradable hydrogel (15% w/v PEGDA) was used so that variability in results associated with a degrading gel could be eliminated. However, once a robust and reproducible protocol was identified, we switched the non-degradable gel with its degradable counterpart for the *in vivo* component of this investigation. This switch was performed so that new cartilage would be able to fill the spaces left behind when PEGDA degraded within in the chondral defects. The PEGDA concentration was kept constant for both *in-vitro* and *in-vivo* studies.

We found a significantly higher shear strength (p<0.05; Fig 2(A)) after 28 days of tissue culture in Group 1 specifically, HCs Agar-HBMSCs PEGDA- No HA constructs in comparison to equivalent constructs with HA. From this observation, we interpret that matrix produced by HCs incorporating HA does not promote chondral-chondral integration; rather, the HA particles aggregate as non-heterogonous components along the interface of the two materials which ultimately results in reduction of shear strength. In contrast, we found a significantly higher shear strength (p<0.05; Fig 2(B)) in HCOAs Agar-HBMSCs PEGDA-HA samples in comparison to its counterpart without HA. Interestingly thus, an opposing effect was observed (compared to HC-derived matrix) in the presence of osteoarthritis wherein the HA did support significant improvement (p < 0.05) in interfacial strength, and hence more effective integration between the two tissue matrices. These findings under a diseased state were consistent with our earlier work on HA-interfacial strength promotion between cartilage and bone matrix [22].

A narrowing of the gap in both HCs Agar-HBMSCs PEGDA- No HA and HCs Agar-HBMSCs PEGDA-HA samples was observed after 28 days of tissue culture (Fig 3). However, in both cases, an intermediate transition zone between the HBMSC and chondrocyte-derived engineered cartilage matrices was absent. We note that a sharper or more abrupt transition between two matrices, i.e., a smaller spatial transition zone is indicative of less effective integration [24].

On the other hand, after 28 days of culture in HCOAs Agar-HBMSCs PEGDA-HA samples, we observed the formation of a thin transition zone (Fig 3). This transition zone was largely occupied by calcium phosphate deposits. Notably, a similar presence of calcium phosphate but in larger quantities is present between cartilage and bone matrix [22]. Subsequent EDS analysis in the transition zone between these tissues quantified elemental Calcium in the order of ∼ 6.41% (Fig 4A). Moreover, EDS mapping (Fig 4B) confirmed the presence of both Calcium and Phosphorus elements, both constituents of HA, thereby confirming the presence of HA within the transition zone. In sum, these findings provide conclusive evidence that the transition zone was created due to the settlement of HA nanoparticles primarily along the interface location.

To further evaluate the effects of a more stable interface via HA incorporation in diseased environments, we conducted a preliminary *in vivo* investigation in the rabbit osteoarthritic knee model. We observed significantly improved (p < 0.05) cellularity in chondral defects that were treated with PEGDA+ HA compared to when HA was not used (Fig 5C). The enhanced stability of PEGDA with the HA incorporation is likely to have promoted migration of bone marrow stem cells after bone microfracture. Hence *in vivo*, we anticipate that engineered cartilage tissues will more effectively integrate with surrounding articular cartilage, not solely due to HA, but also by the accelerated ECM secretion that can now be deposited by a greater number of cells present within the defect.

We emphasize however that the limitations of the explant studies are that it is preliminary and we can for the moment, only conjecture on enhanced stability indirectly based on the higher concentrations of cells within the defect, i.e., more cells will create more ECM, enabling greater tissue filling, hence greater repair and hence, stability. On the other hand the *in vitro* studies by way of the mechanical test resulted in the HA group exhibiting significantly higher (p < 0.05) strength over without-HA controls under osteoarthritic conditions. Note that osteoarthritis was also present in the rabbit model via ligament resection surgery 2 weeks prior to chondral defect creation. Thus direct evidence of HA promoting enhanced stability under osteoarthritic conditions was still assessed and achieved albeit in *in vitro* rather than under *in vivo* environments. Conclusive evidence on enhanced stability *in vivo* can only be determined via interfacial strength testing of joint explants, ideally from a similar study design utilizing larger animal models (e.g. goat), where chondral defects of greater thickness can be created relative to the rabbit model, which is in the order of only a few hundred micron. For the time-being, we have nonetheless demonstrated enhanced mechanical stability of the engineered cartilage using HA in osteoarthritic states from the mechanical testing outcomes.

We observed *in vitro* that in samples without HA, the HBMSC-portion of HCs Agar-HBMSCs PEGDA- No HA constructs exhibited a healthy cartilage phenotype after 28 days of culture (Fig 6(A)). However, when HA was incorporated there was a high expression of the genes, MMP13, Runx2, Collagen Type X and in particular, significant expression of Osteocalcin (p < 0.05) at the proximal region to the interface. The absence of these bone ECM genes thus preserved the cartilage phenotype when HA was not incorporated in healthy engineered cartilage matrix. However in contrast, corresponding HBMSC regions in HCOAs Agar-HBMSCs PEGDA-HA samples, revealed in particular, a significantly lower expression (p< 0.05) of Collagen Type I in the proximal region to the interface, i.e., indicative of a preservation of articular cartilage phenotype in osteoarthritic environments, this time when HA was incorporated (while still maintaining robust expression of Aggrecan, SOX9 and Collagen Type II; Fig 6(B)).

Finally in osteoarthritic environments, in the specimens with HA, distal locations deep within the HBMSC-derived engineered tissue did not or negligibly expressed MMP13, Runx2 and Collagen X (p < 0.05; Fig 6(B)). Recall on the other hand that in healthy, chondrocyte-derived matrix, specimens containing HA expressed these bone-related genes. Ironically thus, the osteoarthritic state of adjacent cartilage matrix is a necessary precursor to permit the presence of calcium phosphate at the interface location, noting that calcium phosphate was found to be absent when the adjacent environment comprised of engineered cartilage derived from HCs (Fig 3(B)). From a clinical perspective, we note that following cartilage injury, osteoarthritis rapidly develops [37–39] and is usually a co-morbidity when small to medium size cartilage defects [40] are present. However, a limitation of this investigation is that the mechanism(s) that lead to greater integration remains unknown. Also uncertain is the extent to which the spatial presence of calcium phosphate in the transition zone is due to HA nanoparticle sedimentation as opposed to calcium phosphate deposition. Nonetheless, taken into the context of the present study, our findings do at minimum suggest that the combination of an osteoarthritic cartilage matrix environment with the presence of HA nanoparticles at the interface promotes enhanced mechanical integration between chondrocyte-derived and HBMSC-derived chondrogenic ECM.

Even though our results appear promising and our HA-based protocol could potentially be transferred directly to clinical photopolymerizable tissue engineered cartilage strategies currently being investigated [19, 41], we note that many of our findings were obtained from *in vitro* experiments. Specifically, we observed an increase in integration shear strength of engineered cartilage with the osteoarthritic cartilage when HA particles were incorporated. However, the critical strength that would be needed clinically for effective long-term engineered cartilage retention in order to permit an adequate amount of time for subsequent tissue regeneration within the defect space has not been identified to date. Nonetheless in the preliminary *in vivo* work that was presented here, it is clear that the presence of HA does augment cellularity within repair tissue that fills the defect space.

## Conclusions

In summary, we were able to demonstrate the utility of HA particles for chondral-chondral integration when the chondrocyte-derived cartilage matrix is osteoarthritic. A calcified cartilage matrix manifests itself between HBMSC and HCOA derived engineered cartilage tissues in the form of a spatial transition zone, thereby forming a stronger and a more stable interface. From an *in vivo* perspective, the increased stability that results from using HA nanoparticles within the hydrogel scaffold is likely to have enhanced the cellularity of *de novo* tissues that filled rabbit chondral defects, four weeks following treatment. This higher presence of cells can be considered important in the context of accelerating long-term cartilage remodeling.

## Supporting Information

S1 File Raw experimental data for cell viability of healthy and osteoarthritic chondrocytes cultured in Agar gels over 28 days. (XLSX)

S2 File Raw experimental data for shear strength for healthy cartilage constructs with HBMSCs in PEGDA with and without HA. (XLSX)

S3 File Raw experimental data for shear strength for osteoarthritis cartilage constructs with HBMSCs in PEGDA with and without HA. (XLSX)

S4 File Additional details on EDS spectra and map analyses for Calcium and Phosphorous elements within the transition zone between engineered cartilage and engineered bone matrix. (DOCX)

S5 File Raw experimental Data for q(Rt-PCR) for HBMSCs derived engineered cartilage integrated to HC-secreted cartilage. (XLSX)

S6 File Raw experimental Data for q(Rt-PCR) for HBMSCs derived engineered cartilage integrated to HCOA-secreted cartilage. (XLSX)
