# Supplementary material for: Integration of Stem Cell to Chondrocyte-Derived Cartilage Matrix in Healthy and Osteoarthritic States in the Presence of Hydroxyapatite Nanoparticles
Source: PLoS One. 2016 Feb 12;11(2):e0149121. doi: 10.1371/journal.pone.0149121 (PMC4752260; doi:10.1371/journal.pone.0149121)
Supplement: S4 File — (DOCX) [file pone.0149121.s004.docx]

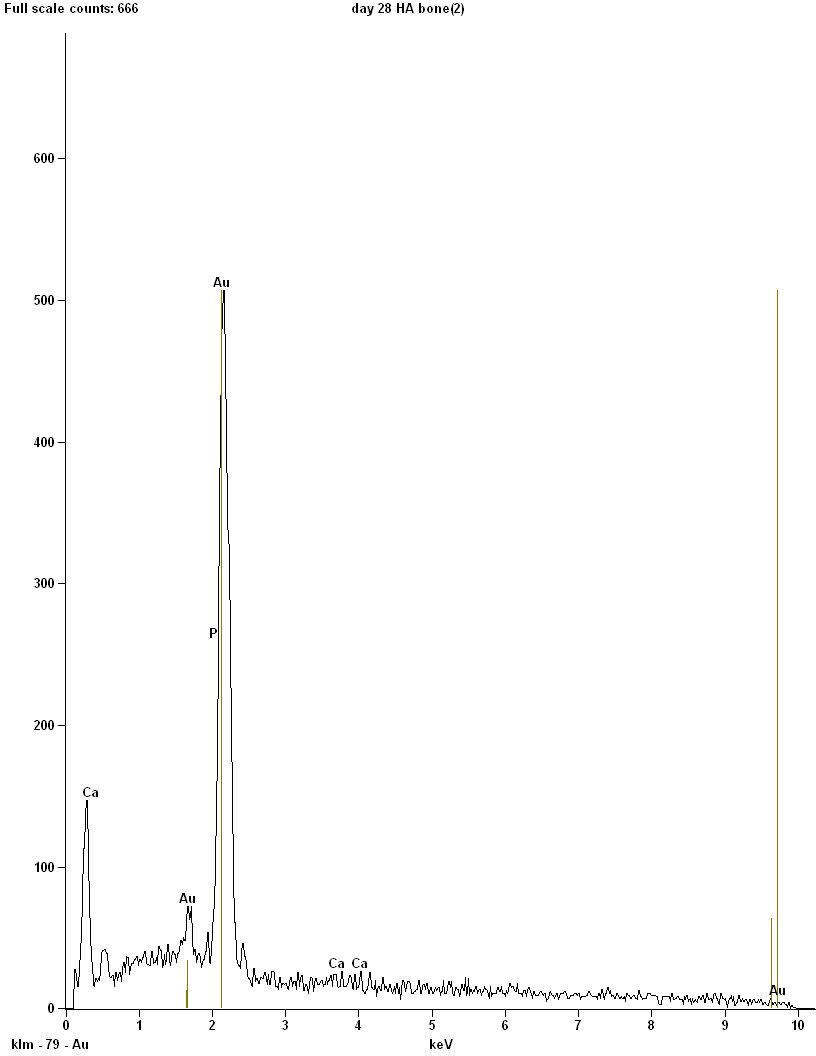


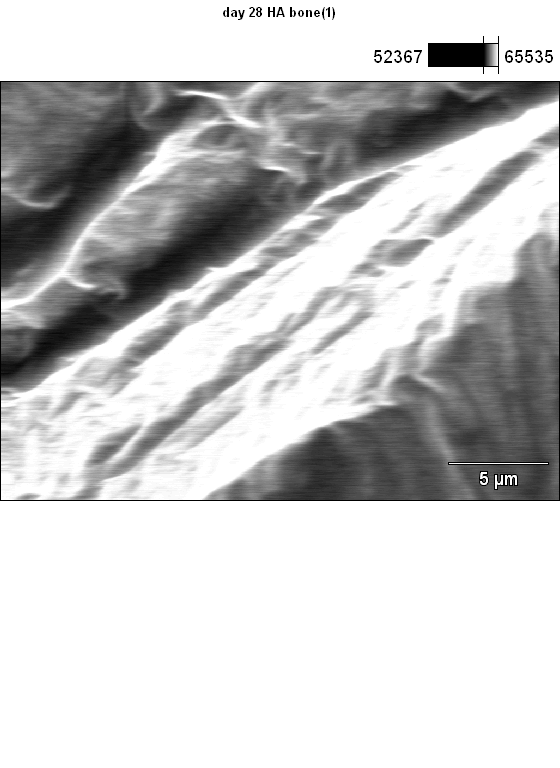


Accelerating Voltage: 10.0 kV Magnification: 4300


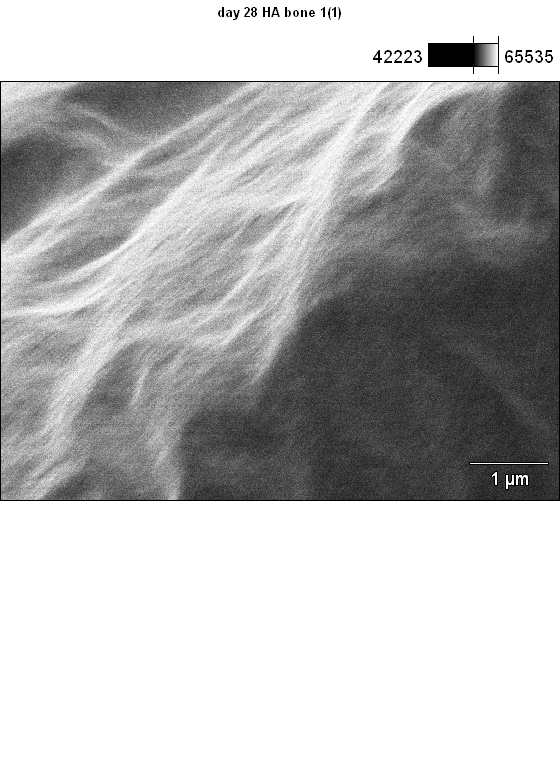


Accelerating Voltage: 10.0 kV Magnification: 17000


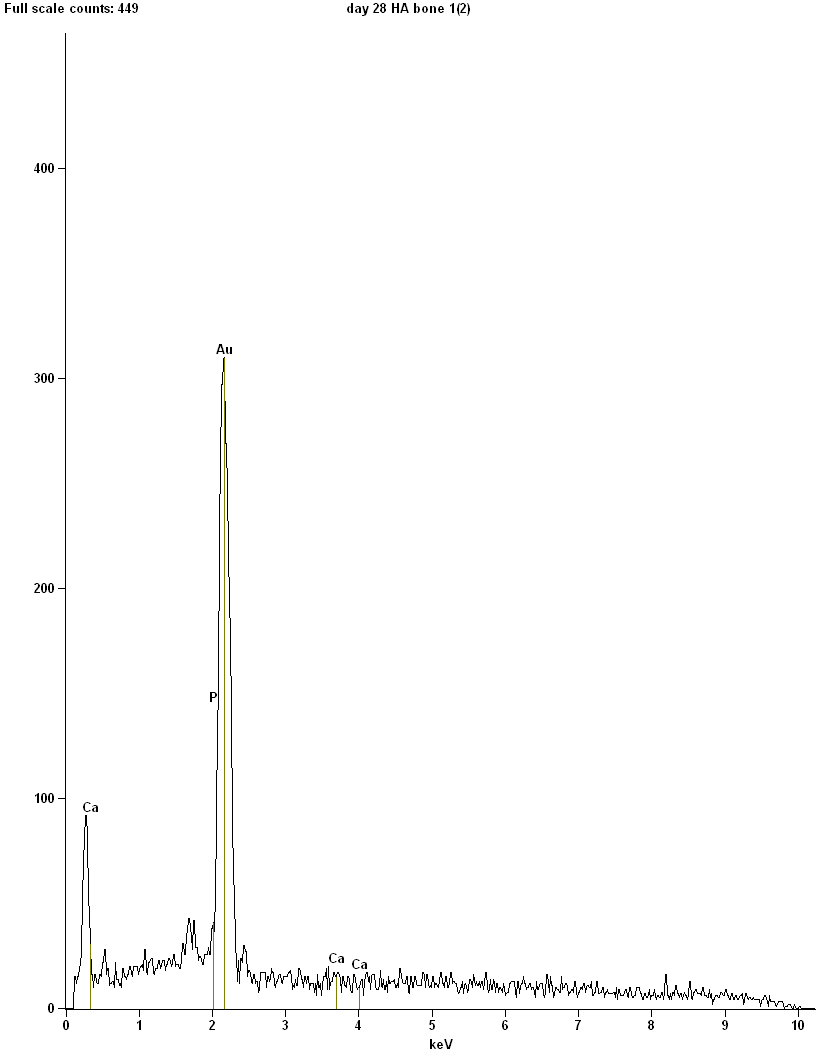


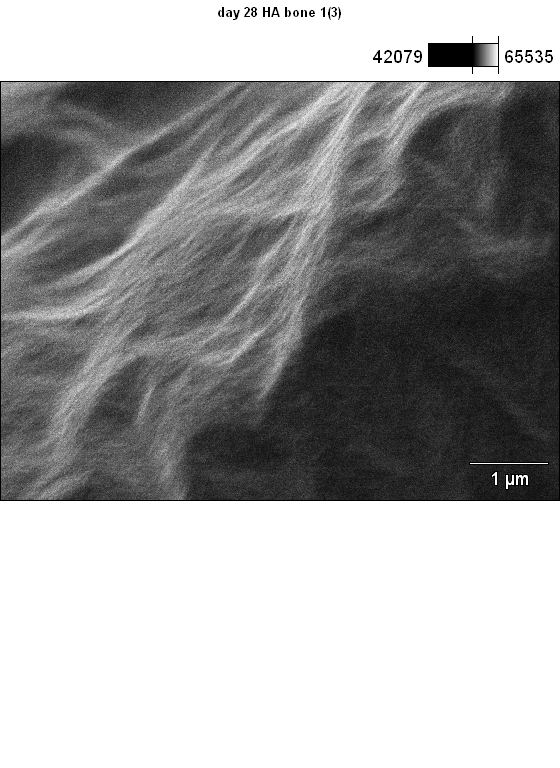


Accelerating Voltage: 10.0 kV Magnification: 17000


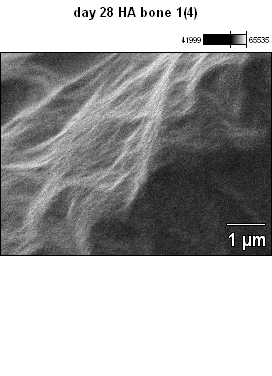

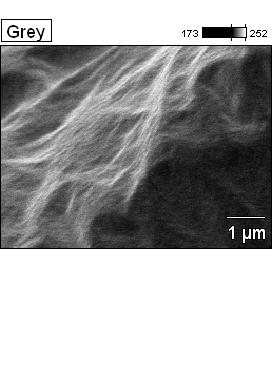


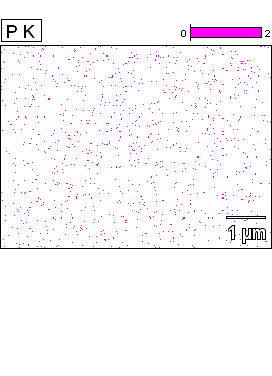

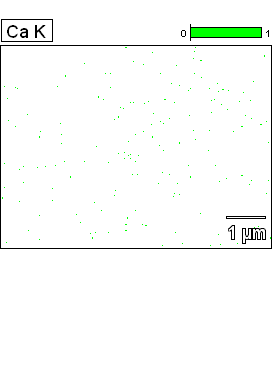


Data Type: Counts Mag: 17000 Acc. Voltage: 10.0 kV


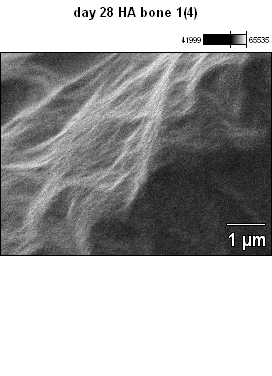

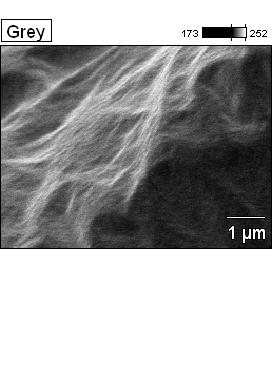


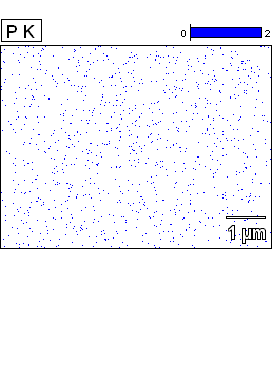

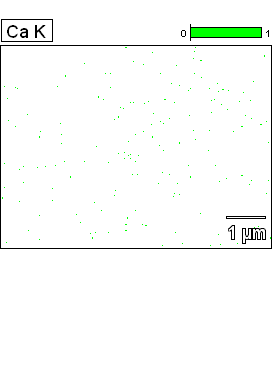


Data Type: Counts Mag: 17000 Acc. Voltage: 10.0 kV


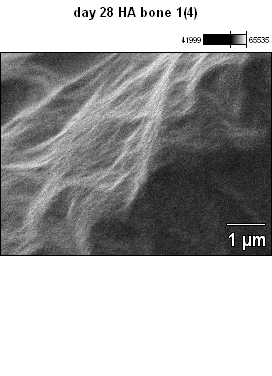

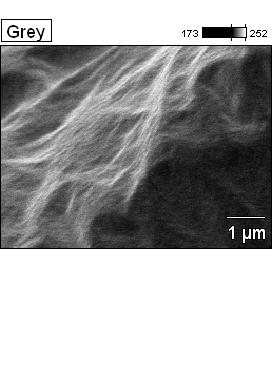


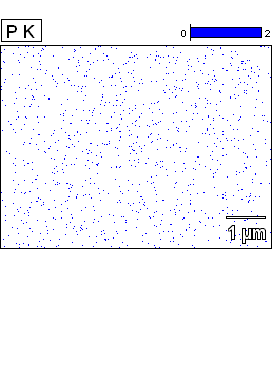

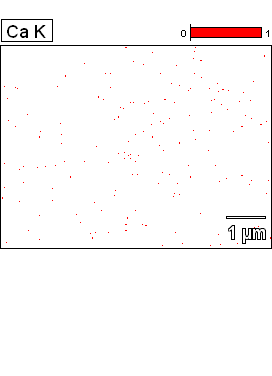


Data Type: Counts Mag: 17000 Acc. Voltage: 10.0 kV


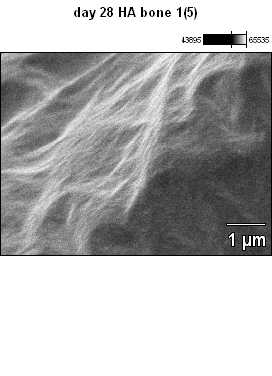

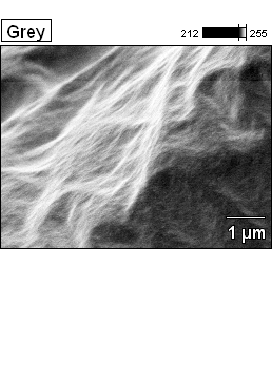


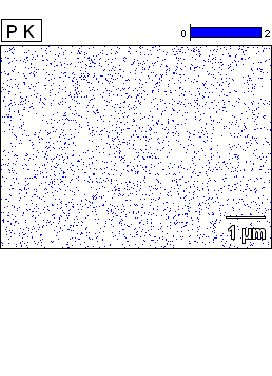

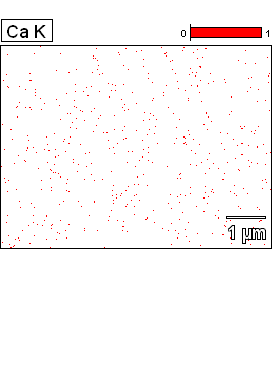


Data Type: Counts Mag: 17000 Acc. Voltage: 10.0 kV
